# Potential of Thiocolchicoside as an Antimicrobial Repurposing Agent: An In Vitro Study

**DOI:** 10.7759/cureus.110122

**Published:** 2026-06-02

**Authors:** Akash V Devi, Chitra Khanwelkar

**Affiliations:** 1 Pharmacology, Krishna Institute of Medical Sciences, Krishna Vishwa Vidyapeeth (Deemed to be University), Karad, IND

**Keywords:** drug repurposing, gram-negative bacteria, gram-positive bacteria, minimum inhibitory concentration (mic), thiocolchicoside

## Abstract

Antimicrobial resistance (AMR) is a global health emergency that poses a hazard to the effective management of infectious diseases. Drug repurposing provides a cost-effective and expeditious solution by identifying new applications for existing pharmaceuticals. A semi-synthetic derivative of colchicoside, thiocolchicoside (THC), has been recently investigated for its potential anticancer and antimicrobial properties. It is prescribed as a muscle relaxant. THC was tested against a variety of gram-positive and gram-negative bacteria, as well as fungal isolates, at concentrations ranging from 4000 to 62.5 µg/mL. Standard antibacterial and antifungal controls were provided by gentamicin (GN) and fluconazole (FZ). Turbidity and absorbance-based assays at 600 nm were employed to ascertain the minimum inhibitory concentrations (MICs). THC demonstrated MICs of 125 µg/mL against *Streptococcus mutans, Staphylococcus aureus, Lactobacillus acidophilus, Escherichia coli, *and* Candida albicans*. The moderate antimicrobial activity with variable potency was indicated by the higher MICs for *Pseudomonas aeruginosa* (1000 µg/mL), *Proteus mirabilis* (1000 µg/mL), *Klebsiella pneumoniae* (500 µg/mL), and *Aspergillus niger* (250 µg/mL). Particularly for *Aspergillus niger*, fungal inhibition necessitated elevated dosages. The results were presented as the mean ± standard deviation, and all experiments were conducted in triplicate. Statistically significant differences (p < 0.05) were identified in the MIC values of THC compared to standard drugs GN and FZ across identical microbiological strains utilizing paired t-tests. THC exhibited lower activity compared to GN and FZ, yet it showed initial promise as an adjunct antimicrobial agent; nonetheless, this assessment is tentative due to the comparatively elevated MIC values and the constraints of in vitro data lacking mechanistic or in vivo corroboration. In order to improve its therapeutic profile, additional mechanistic studies, synergistic evaluations, and nanotechnology-based formulations are necessary.

## Introduction

Drug repurposing is a promising approach that expedites clinical translation and reduces development time and cost by identifying new therapeutic applications for existing drugs [1,2]. This method has effectively uncovered novel applications for a variety of non-antibiotic agents, such as anticancer and anti-inflammatory drugs. This has expanded the therapeutic arsenal against drug-resistant infections and has assisted in the mitigation of antibiotic crises caused by the misuse of current drugs [3,4]. Thiocolchicoside (THC), a semi-synthetic derivative of colchicoside, is conventionally prescribed as a muscle relaxant for musculoskeletal disorders. Its pharmacological activity is primarily mediated through the modulation of the gamma-aminobutyric acid type-A (GABA-A) receptor [5,6]. THC is often used as a muscle relaxant; nevertheless, preliminary research indicates potential anticancer and antibacterial properties [7-10]. The antibacterial potential has been predominantly examined by in vitro experiments, demonstrating activity against both gram-positive and gram-negative bacteria, as well as fungi, with moderate minimum inhibitory concentration (MIC) values observed. Moreover, nanogel formulations containing THC have been evaluated for their antibacterial and cytotoxic properties in laboratory environments [8]. The findings are tentative and primarily confined to experimental models; however, they support the repurposing of THC as an adjunctive drug, considering its recognized clinical safety profile and emerging evidence of multi-target efficacy. Antimicrobial resistance (AMR) remains a global health issue, as treatment options are restricted by the increasing resistance to common antibiotics, including aminoglycosides and β-lactams [11-13]. Repurposing existing compounds provides a rapid and cost-effective method of identifying novel antimicrobial agents in light of this imperative need. The assessment of THC as a potential adjuvant therapy in light of increasing AMR is bolstered by preliminary evidence of its anticancer and antibacterial properties. Previous research has predominantly evaluated THC in vitro, including testing against gram-positive and gram-negative bacteria and fungi, revealing reasonable MIC values. Moreover, nanogel formulations containing THC have exhibited antibacterial and cytotoxic properties in vitro [8]. Although these data are preliminary and generate hypotheses, they justify the repurposing of THC due to its recognized clinical safety as a muscle relaxant and its developing multi-target potential. In the present age of increasing AMR, drug repurposing provides a practical method to discover novel therapeutic uses for existing drugs with known safety profiles. THC, typically utilized as a muscle relaxant, has demonstrated limited antibacterial action in preclinical tests. These findings, however constrained, offer a justification for additional investigation. This study was conducted as an exploratory and comparative assessment of THC's antibacterial and antifungal efficacy against specific reference strains, using gentamicin (GN) and fluconazole (FZ) as standard comparators.

## Materials and methods

Chemicals and reagents 

THC (Batch No. RPAD0152; CAS No. 602‑41‑5; PubChem CID: 9915886) was procured from Dr. Reddy's Laboratories Pvt. Ltd., Hyderabad, India. The compound was dissolved in sterile distilled water to prepare a stock solution of 4 mg/mL prior to serial dilution. The standard antibacterial control was GN sulphate (HiMedia Laboratories Private Limited, Thane, Maharashtra, India; Product Code CMS461‑5G; CAS No. 1405‑41‑0; EC No. 215‑778‑9). The standard antifungal control was FZ (HiMedia Laboratories Private Limited, Thane, Maharashtra, India; Product Code CMS8387; CAS No. 86386‑73‑4). Analytical-grade culture media were procured from HiMedia Laboratories Private Limited, Thane, Maharashtra, India, which includes Nutrient Broth (NB; Product Code M002‑500G) and Sabouraud Dextrose Agar (SDA; Product Code M063). Both GN and FZ were dissolved in sterile distilled water to prepare a stock solution for the serial dilution assay.

Microorganisms

The antimicrobial activity was assessed against the reference strains *Escherichia coli *(ATCC 8739)*, Staphylococcus aureus *(ATCC 6538)*, Pseudomonas aeruginosa *(ATCC 15442)*, Streptococcus mutans *(ATCC 25175)*, Lactobacillus acidophilus *(ATCC 4536)*, Proteus mirabilis *(ATCC 4630)*, Klebsiella pneumoniae *(ATCC 13883)*, Candida albicans *(ATCC 14053)*, *and* Aspergillus niger *(ATCC 11414)* *as these strains are extensively utilized in susceptibility testing and represent therapeutically significant pathogens.

Culture preparation

The inocula were adjusted to a standardized concentration of 2 × 10⁴ cells/mL after the cell density was determined using a hemocytometer [14]. Bacterial inocula were developed by suspending colonies in sterile saline and calibrating turbidity to the 0.5 McFarland standard (~1 × 10⁸ CFU/mL) with a densitometer, before performing a serial dilution assay.

Serial dilution assay

The broth dilution method was employed to ascertain MICs following the European Committee on Antimicrobial Susceptibility Testing (EUCAST) 2003 criteria [14]. Serial dilutions of THC, GN, and FZ were developed in nutritional broth at concentrations between 4000 and 62.5 µg/mL (Table 1). Positive controls comprised GN for bacterial strains and FZ for fungal strains, while negative controls consisted of broth containing solely the solvent at the identical final concentration utilized in the test tubes. Growth was evaluated by optical density (OD) measured at absorbance of 600 nanometer (OD600) measurements, and inhibition (%) was computed using the following formula: (absorbance of control - absorbance of sample) divided by absorbance of control, multiplied by 100. MIC endpoints were established utilizing OD thresholds: ≤0.29 (no growth), 0.30-0.69 (partial growth), and ≥0.70 (substantial growth). These criteria were uniformly implemented across all strains in triplicate experiments. Bacterial cultures were incubated at 37°C for 48 hours, while fungal inocula were subjected to species-specific conditions (*Candida albicans* at 35 ± 2°C for 24-48 hours; *Aspergillus niger *at 28 ± 2°C for 48-72 hours), in accordance with the Clinical and Laboratory Standards Institute (CLSI)/EUCAST recommendations. 

**Table 1:** Concentration range of standard drugs GN and FZ and test drug THC used for antibiotic susceptibility testing on selected organisms GN: gentamicin; THC: thiocolchicoside; FZ: fluconazole; µg/mL: microgram per millilitre

Organism tested	GN (µg/mL)	THC (µg/mL)	FZ (µg/mL)
Escherichia coli	4000-62.5	4000-62.5	4000-62.5
Staphylococcus aureus	4000-62.5	4000-62.5	4000-62.5
Pseudomonas aeruginosa	4000-62.5	4000-62.5	4000-62.5
Streptococcus mutans	4000-62.5	4000-62.5	4000-62.5
Lactobacillus acidophilus	4000-62.5	4000-62.5	4000-62.5
Klebsiella pneumoniae	4000-62.5	4000-62.5	4000-62.5
Proteus mirabilis	4000-62.5	4000-62.5	4000-62.5
Candida albicans	4000-62.5	4000-62.5	4000-62.5
Aspergillus niger	4000-62.5	4000-62.5	4000-62.5

Statistical analysis 

Data was evaluated utilizing descriptive statistics (mean ± SD). Comparisons were conducted using the paired Student's t-test, with pairs defined as MIC values for THC versus GN in bacteria and THC versus FZ in fungus for the same organisms. Assumptions for parametric analysis, such as normality and homogeneity of variance, were validated before testing. Due to the features of the dataset, neither regression analysis nor post hoc analysis was used. Statistical analysis was performed with GraphPad InStat version 3.06 (GraphPad Software, San Diego, CA, USA). A p-value of less than 0.0001 was deemed statistically significant.

## Results

OD600 values for gram-positive organisms were assessed at concentrations from 4000 to 62.5 µg/mL. Results are presented as mean ± standard deviation (SD) from triplicate experiments, alongside computed percentage inhibition and MIC values. The data exhibit a distinct concentration-dependent inhibitory action, with GN consistently yielding lower OD values and higher inhibition percentages than THC. Table 2 delineates the comparative results for *Staphylococcus aureus, Streptococcus mutans, *and *Lactobacillus acidophilus*, offering a consistent quantitative evaluation of antimicrobial efficacy across the examined concentration spectrum. 

**Table 2:** Mean OD ± SD, percentage inhibition, and MIC values for Staphylococcus aureus, Streptococcus mutans, and Lactobacillus acidophilus at concentrations 4000-62.5 µg/mL comparing THC and GN OD: optical density; SD: standard deviation; MIC: minimum inhibitory concentration; µg/mL: microgram per millilitre; THC: thiocolchicoside; GN: gentamicin

Concentration (µg/mL)	*Staphylococcus aureus* (mean OD ± SD, % inhibition)	MIC (µg/mL)	*Streptococcus mutans* (mean OD ± SD, % inhibition)	MIC (µg/mL)	*Lactobacillus acidophilus* (mean OD ± SD, % inhibition)	MIC (µg/mL)
4000	GN 0.10 ± 0.01, 87.5; THC 0.26 ± 0.02, 68.75	-	GN 0.11 ± 0.01, 87.50; THC 0.26 ± 0.01, 69.53	-	GN 0.13 ± 0.02, 85.88; THC 0.24 ± 0.01, 71.76	-
2000	GN 0.14 ± 0.01, 82.5; THC 0.31 ± 0.01, 61.25	-	GN 0.15 ± 0.01, 82.42; THC 0.29 ± 0.01, 66.01	-	GN 0.14 ± 0.02, 83.53; THC 0.29 ± 0.02, 67.05	-
1000	GN 0.17 ± 0.01, 78.75; THC 0.35 ± 0.01, 56.25	GN 62.5; THC 125	GN 0.18 ± 0.01, 78.90; THC 0.33 ± 0.01, 61.32	GN 62.5; THC 125	GN 0.19 ± 0.02, 77.65; THC 0.33 ± 0.02, 62.35	GN 62.5; THC 125
500	GN 0.20 ± 0.01, 75.00; THC 0.39 ± 0.02, 51.25	-	GN 0.24 ± 0.02, 71.25; THC 0.35 ± 0.01, 56.25	-	GN 0.22 ± 0.01, 75.29; THC 0.37 ± 0.01, 56.47	-
250	GN 0.21 ± 0.02, 71.25; THC 0.43 ± 0.02, 46.25	-	GN 0.29 ± 0.02, 65.58; THC 0.45 ± 0.03, 47.65	-	GN 0.26 ± 0.02, 71.76; THC 0.44 ± 0.03, 48.23	-
125	GN 0.23 ± 0.02, 67.5; THC 0.46 ± 0.01, 42.05	-	GN 0.36 ± 0.01, 57.81; THC 0.50 ± 0.01, 41.40	-	GN 0.32 ± 0.01, 64.71; THC 0.48 ± 0.02, 44.70	-
62.5	GN 0.25 ± 0.01, 61.25; THC 0.52 ± 0.01, 35.00	-	GN 0.39 ± 0.01, 54.29; THC 0.53 ± 0.02, 37.5	-	GN 0.36 ± 0.02, 57.65; THC 0.52 ± 0.01, 38.82	-

**Figure 1:**
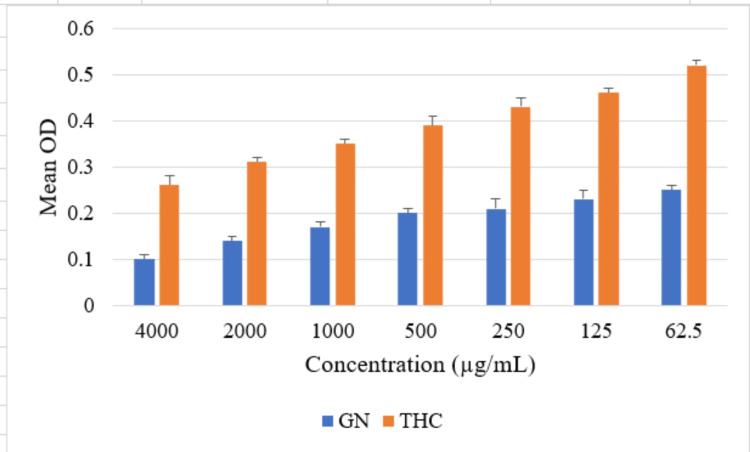
Comparative OD (mean OD ± SD) of Staphylococcus aureus exposed to THC versus GN across concentrations 4000-62.5 µg/mL OD: optical density; SD: standard deviation; µg/mL: microgram per millilitre; THC: thiocolchicoside; GN: gentamicin

**Figure 2:**
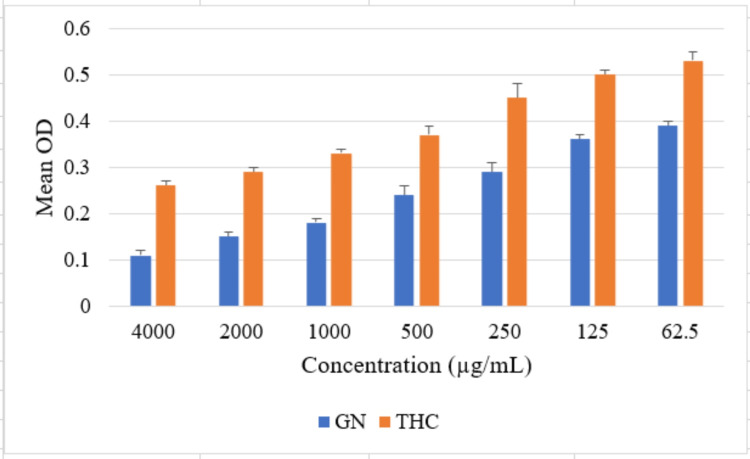
Comparative OD (mean OD ± SD) of Streptococcus mutans exposed to THC versus GN across concentrations 4000-62.5 µg/mL OD: optical density; SD: standard deviation; µg/mL: microgram per millilitre; THC: thiocolchicoside; GN: gentamicin

**Figure 3:**
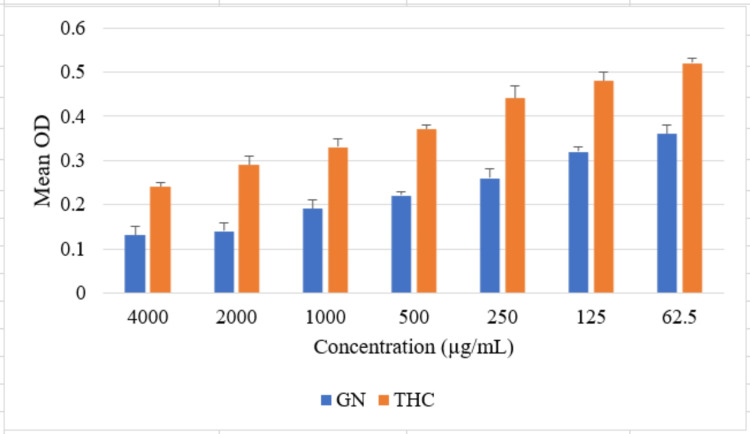
Comparative OD (mean OD ± SD) of Lactobacillus acidophilus exposed to THC versus GN across concentrations 4000-62.5 µg/mL OD: optical density; SD: standard deviation; µg/mL: microgram per millilitre; THC: thiocolchicoside; GN: gentamicin

Figures 1-3 illustrate the OD600 values ± SD derived from triplicate studies for gram-positive organisms. The graphs illustrate a distinct concentration-dependent inhibitory effect, with GN consistently exhibiting lower OD values and elevated inhibition percentages relative to THC. These visual trends enhance the quantitative results in Table 2, offering a consistent comparison of antimicrobial activity over the examined concentration range.

OD600 values for gram-negative organisms were analyzed at concentrations from 4000 to 62.5 µg/mL. Results are presented as mean ± SD from triplicate experiments, along with computed percentage inhibition and MIC values. The observations underscore the relative inhibitory effects of THC and GN, with GN consistently exhibiting lower OD values and more pronounced inhibition at most concentrations. Table 3 delineates the concentration-specific results for *Escherichia coli* and *Pseudomonas aeruginosa*, whereas Table 4 encapsulates the data for *Klebsiella pneumoniae* and *Proteus mirabilis*. This systematic presentation facilitates reliable intra-organism comparisons and highlights the heterogeneity between the two treatments.

**Table 3:** Mean OD ± SD, percentage inhibition, and MIC values for Escherichia coli and Pseudomonas aeruginosa at concentrations 4000-62.5 µg/mL comparing THC and GN OD: optical density; SD: standard deviation; µg/mL: microgram per millilitre; THC: thiocolchicoside; GN: gentamicin; MIC: minimum inhibitory concentration

Concentration (µg/mL)	*Escherichia coli* (mean OD ± SD, % inhibition)	MIC (µg/mL)	*Pseudomonas aeruginosa *(mean OD ± SD, % inhibition)	MIC (µg/mL)
4000	GN 0.13 ± 0.01, 84.14; THC 0.21 ± 0.02, 74.39	-	GN 0.12 ± 0.01, 85.54; THC 0.24 ± 0.03, 71.08	-
2000	GN 0.15 ± 0.01, 81.70; THC 0.26 ± 0.01, 68.29	-	GN 0.15 ± 0.01, 82.32; THC 0.36 ± 0.01, 56.62	-
1000	GN 0.20 ± 0.01, 75.60; THC 0.32 ± 0.02, 60.97	GN 62.5; THC 125	GN 0.19 ± 0.02, 76.70; THC 0.46 ± 0.01, 44.57	GN 62.5; THC 1000
500	GN 0.23 ± 0.02, 71.95; THC 0.37 ± 0.01, 54.87	-	GN 0.23 ± 0.01, 72.28; THC 0.51 ± 0.01, 38.55	-
250	GN 0.27 ± 0.01, 67.07; THC 0.42 ± 0.01 48.78	-	GN 0.27 ± 0.02, 67.06; THC 0.55 ± 0.02, 33.73	-
125	GN 0.31 ± 0.02, 63.41; THC 0.47 ± 0.01, 43.90	-	GN 0.32 ± 0.02, 61.44; THC 0.62 ± 0.02, 25.70	-
62.5	GN 0.36 ± 0.01, 56.09; THC 0.52 ± 0.02, 37.80	-	GN 0.37 ± 0.01, 55.42; THC 0.67 ± 0.02, 20.48	-

**Table 4:** Mean OD ± SD, percentage inhibition, and MIC values for Klebsiella pneumoniae and Proteus mirabilis at concentrations 4000-62.5 µg/mL comparing THC and GN OD: optical density; SD: standard deviation; MIC: minimum inhibitory concentration; µg/mL: microgram per millilitre; THC: thiocolchicoside; GN: gentamicin

Concentration (µg/mL)	*Klebsiella pneumoniae* (mean OD ± SD, % inhibition)	MIC (µg/mL)	*Proteus mirabilis *(mean OD ± SD, % inhibition)	MIC (µg/mL)
4000	GN 0.13 ± 0.04, 85.44; THC 0.36 ± 0.01, 58.62	-	GN 0.21 ± 0.01, 76.13; THC 0.31 ± 0.06, 64.39	-
2000	GN 0.20 ± 0.02, 77.39; THC 0.40 ± 0.01, 54.02	-	GN 0.26 ± 0.01, 70.45; THC 0.47 ± 0.02, 46.96	-
1000	GN 0.25 ± 0.01, 71.26; THC 0.44 ± 0.02, 49.04	GN 62.5; THC 500	GN 0.34 ± 0.02, 60.98; THC 0.52 ± 0.03, 40.90	GN 62.5; THC 1000
500	GN 0.30 ± 0.01, 65.52; THC 0.48 ± 0.01, 44.83	-	GN 0.37 ± 0.02, 58.33; THC 0.57 ± 0.01, 35.22	-
250	GN 0.34 ± 0.02, 60.54; THC 0.54 ± 0.02, 38.31	-	GN 0.40 ± 0.01, 54.54; THC 0.62 ± 0.02, 29.92	-
125	GN 0.41 ± 0.01, 52.87; THC 0.60 ± 0.01, 31.03	-	GN 0.43 ± 0.01, 51.13; THC 0.68 ± 0.01, 22.72	-
62.5	GN 0.44 ± 0.02, 49.04; THC 0.66 ± 0.03, 24.52	-	GN 0.47 ± 0.02, 46.21; THC 0.71 ± 0.01, 19.31	-

**Figure 4:**
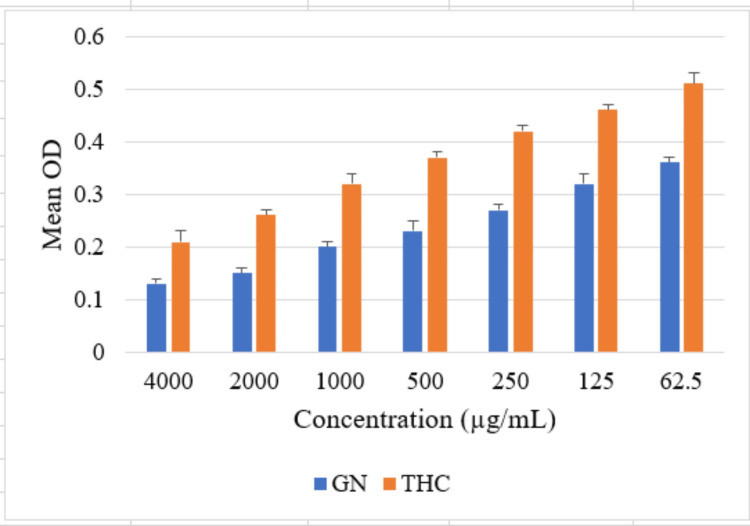
Comparative OD (mean OD ± SD) of Escherichia coli exposed to THC versus GN across concentrations 4000-62.5 µg/mL OD: optical density; SD: standard deviation; µg/mL: microgram per millilitre; THC: thiocolchicoside; GN: gentamicin

**Figure 5:**
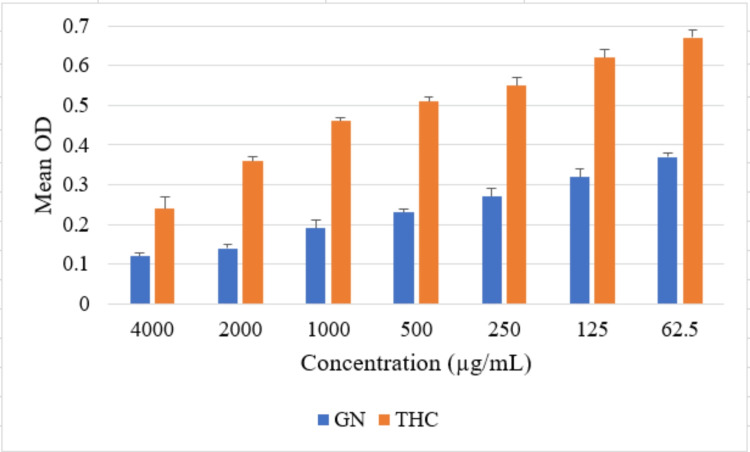
Comparative OD (mean OD ± SD) of Pseudomonas aeruginosa exposed to THC versus GN across concentrations 4000-62.5 µg/mL OD: optical density; SD: standard deviation; µg/mL: microgram per millilitre; THC: thiocolchicoside; GN: gentamicin

Figure 4 and Figure 5 exhibit the OD600 values ± SD derived from triplicate studies for two gram-negative bacteria, *Escherichia coli* and *Pseudomonas aeruginosa*. The graphs illustrate a distinct concentration-dependent inhibitory effect, with GN consistently yielding lower OD values and higher inhibition percentages than THC. These visual trends enhance the quantitative results shown in Table 3, offering a replicable comparison of antimicrobial activity over the evaluated concentration spectrum.

**Figure 6:**
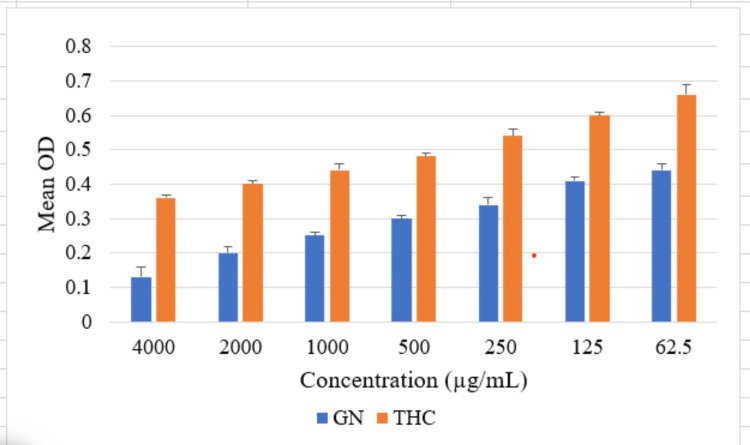
Comparative OD (mean OD ± SD) of Klebsiella pneumoniae exposed to THC versus GN across concentrations 4000-62.5 µg/mL OD: optical density; SD: standard deviation; THC: thiocolchicoside; GN: gentamicin; µg/mL: microgram per millilitre

**Figure 7:**
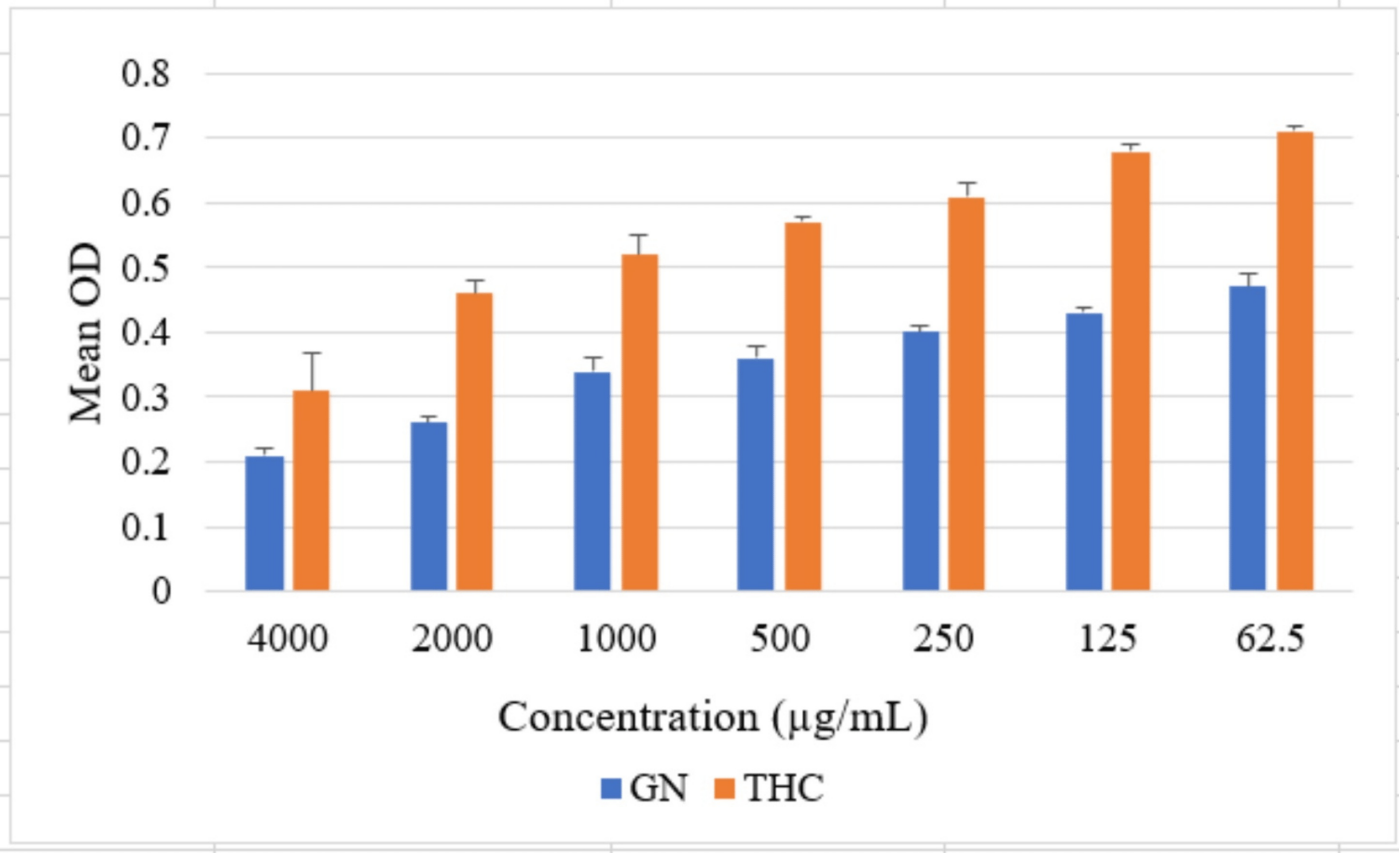
Comparative OD (mean OD ± SD) of Proteus mirabilis exposed to THC versus GN across concentrations 4000-62.5 µg/mL OD: optical density; SD: standard deviation; THC: thiocolchicoside; GN: gentamicin; µg/mL: microgram per millilitre

Figure 6 and Figure 7 show the mean OD600 values ± SD obtained from triplicate studies for two gram-negative organisms, *Klebsiella pneumoniae* and *Proteus mirabilis*. The graphs illustrate a concentration-dependent inhibitory effect, with GN consistently yielding lower OD values and higher inhibition percentages than THC. These visual results enhance the quantitative data shown in Table 4, offering a consistent comparison of antimicrobial efficacy over the examined concentration range.

OD600 values for fungal organisms were assessed at concentrations from 4000 to 62.5 µg/mL. Results are presented as mean ± SD from triplicate experiments, along with computed percentage inhibition and MIC values. The data underscore the relative inhibitory effects of FZ and THC, with FZ consistently yielding lower OD values and elevated inhibition percentages throughout the majority of doses. Table 5 delineates the results for *Candida albicans* and *Aspergillus niger,* offering a consistent quantitative evaluation of antifungal efficacy across the examined concentration spectrum.

**Table 5:** Mean OD ± SD, percentage inhibition, and MIC values for fungi Candida albicans and Aspergillus niger at concentrations 4000-62.5 µg/mL comparing THC and FZ OD: optical density; SD: standard deviation; MIC: minimum inhibitory concentration; µg/mL: microgram per millilitre; THC: thiocolchicoside; FZ: fluconazole

Concentration (µg/mL)	*Candida albicans* (mean ± SD, % inhibition)	MIC (µg/mL)	*Aspergillus niger* (mean ± SD, % inhibition)	MIC (µg/mL)
4000	FZ 0.11 ± 0.02, 86.58; THC 0.26 ± 0.02, 68.29	-	FZ 0.13 ± 0.01, 84.14; THC 0.24 ± 0.02, 70.75	-
2000	FZ 0.15 ± 0.01, 81.70; THC 0.30 ± 0.01, 63.41	-	FZ 0.17 ± 0.01, 80.48; THC 0.28 ± 0.02, 65.85	-
1000	FZ 0.18 ± 0.01, 78.04; THC 0.35 ± 0.01, 53.65	FZ 62.5; THC 125	FZ 0.20 ± 0.01, 75.60; THC 0.35 ± 0.01, 57.31	FZ 62.5; THC 250
500	FZ 0.23 ± 0.01, 71.95; THC 0.38 ± 0.01, 54.03	-	FZ 0.23 ± 0.01, 71.95; THC 0.39 ± 0.01, 52.43	-
250	FZ 0.28 ± 0.01, 65.85; THC 0.43 ± 0.02, 48.78	-	FZ 0.26 ± 0.02, 68.29; THC 0.44 ± 0.02, 47.56	-
125	FZ 0.34 ± 0.03, 58.53; THC 0.49 ± 0.01, 40.24	-	FZ 0.33 ± 0.03, 59.75; THC 0.50 ± 0.02, 39.02	-
62.5	FZ 0.40± 0.01, 51.21; THC 0.54 ± 0.02, 35.36	-	FZ 0.42 ± 0.02, 50.00; THC 0.56 ± 0.01, 31.70	-

**Figure 8:**
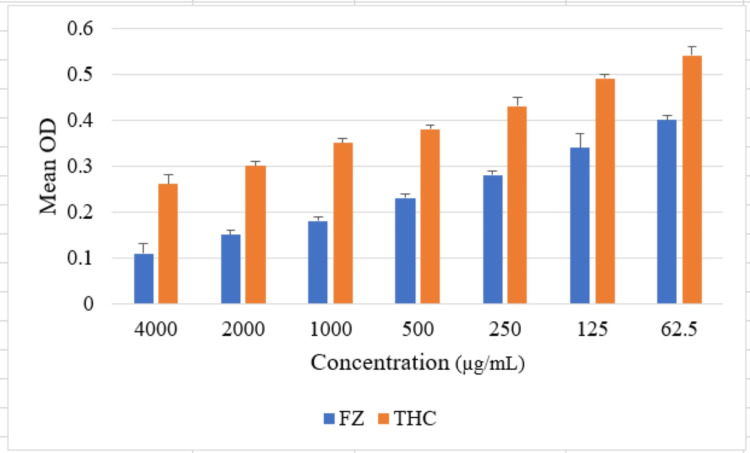
Comparative OD (mean OD ± SD) of Candida albicans exposed to THC versus FZ across concentrations 4000-62.5 µg/mL OD: optical density; SD: standard deviation; THC: thiocolchicoside; FZ: fluconazole; µg/mL: microgram per millilitre

**Figure 9:**
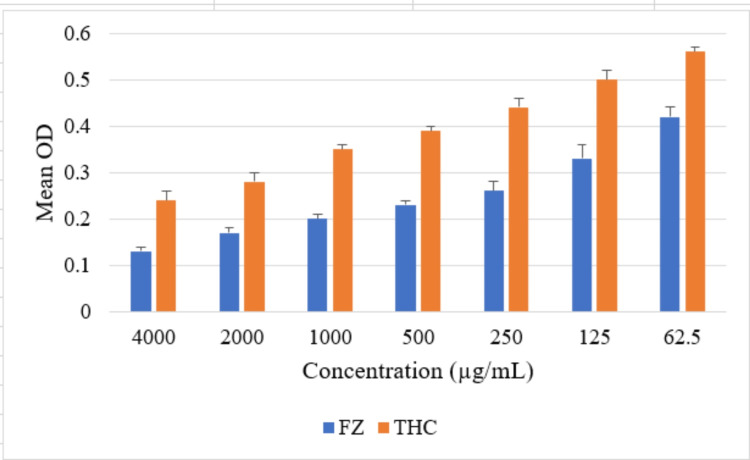
Comparative OD (mean OD ± SD) of Aspergillus niger exposed to THC versus FZ across concentrations 4000-62.5 µg/mL OD: optical density; SD: standard deviation; THC: thiocolchicoside; FZ: fluconazole; µg/mL: microgram per millilitre

OD600 values for fungal species were assessed throughout doses from 4000 to 62.5 µg/mL. Results are presented as mean ± SD from triplicate experiments, along with computed percentage inhibition and MIC values. The data emphasizes the relative inhibitory effects of FZ and THC, with FZ consistently showing lower OD values and elevated inhibition percentages across the majority of doses. Table 5 encapsulates the results for *Candida albicans* and *Aspergillus niger*, whereas Figure 8 and Figure 9 furnish the relevant graphical depictions. Collectively, these findings provide a reliable quantitative and visual evaluation of antifungal efficacy across the examined concentration spectrum.

Table 6 shows the outcomes of paired t-test studies contrasting the inhibitory effects of THC compared to standard reference drugs (GN for bacteria and FZ for fungi). The t-value, p-value, 95% confidence interval of the mean difference, and mean difference in inhibition are provided for each organism. All comparisons revealed highly significant differences (p < 0.0001), with GN and FZ consistently exhibiting stronger inhibition than THC. The results statistically confirm the concentration-dependent inhibitory patterns depicted in Tables 1-5 and Figures 1-9.

**Table 6:** Statistical comparison of THC versus standard drugs across bacterial and fungal organisms THC: thiocolchicoside; GN: gentamicin; FZ: fluconazole

Organism	Comparison	t‑value	P‑value	95% CI of the mean difference	Mean difference in inhibition
Staphylococcus aureus	GN vs THC	23.290	<0.0001	20.833-25.724	23.279
Streptococcus mutans	GN vs THC	42.037	<0.0001	15.888-17.852	16.870
Lactobacillus acidophilus	GN vs THC	15.131	<0.0001	15.220-21.092	18.156
Escherichia coli	GN vs THC	12.152	<0.0001	12.659-19.043	15.851
Pseudomonas aeruginosa	GN vs THC	10.441	<0.0001	22.972-37.037	30.004
Klebsiella pneumoniae	GN vs THC	30.027	<0.0001	21.216-24.981	23.099
Proteus mirabilis	GN vs THC	10.877	<0.0001	17.532-27.711	22.621
Candida albicans	FZ vs THC	18.122	<0.0001	16.076-21.095	18.586
Aspergillus niger	FZ vs THC	16.448	<0.0001	15.272-20.611	17.941

## Discussion

This study revealed that THC displays limited antibacterial and antifungal efficacy, with MIC values regularly above those of conventional drugs like GN and FZ. Although measurable inhibition was noted against several organisms, its efficacy was constrained in comparison to traditional pharmaceuticals, and the comparatively high MIC values indicate selective rather than broad-spectrum activity. Inhibition was more significant in gram-positive bacteria (*Lactobacillus acidophilus, Streptococcus mutans, Staphylococcus aureus*) and the fungus *Candida albicans*, while gram-negative bacteria (*Escherichia coli, Pseudomonas aeruginosa, Proteus mirabilis, Klebsiella pneumoniae*) and the fungus *Aspergillus niger* necessitated elevated concentrations, indicating reduced susceptibility. Differences particular to organisms were apparent. This study did not empirically investigate the proposed mechanisms of resistance and susceptibility, such as changes in cell wall composition, efflux pump functionality, and intrinsic resistance. These explanations are presented as hypotheses to clarify the observed variability, rather than as mechanistic discoveries, and are corroborated by previous publications on efflux pump-mediated resistance [15,16]. Drug repurposing has attracted interest as a technique to combat the worldwide AMR challenge, utilizing proven safety profiles to accelerate clinical translation [3,17]. Historically utilized as a muscle relaxant, THC is currently being evaluated for its additional antibacterial properties. Their efficiency against *Pseudomonas aeruginosa* and *Proteus mirabilis* is particularly significant due to their link with multidrug-resistant illnesses [18,19]. Although THC requires relatively high concentrations to exhibit action, its efficacy may be enhanced through structural alterations or advanced delivery systems; yet, this remains a theory that necessitates further investigation. Although THC requires comparatively elevated doses to exhibit antibacterial effectiveness, its overall safety profile for such uses is still unconfirmed, and the need for greater dosages may increase treatment costs. The limitations highlight the preliminary nature of the findings and suggest that future research should explore structural alterations or advanced delivery methods to enhance efficacy within safe therapeutic boundaries. Nanotechnology-based formulations, including nanogels and nanoparticles, have shown efficacy in enhancing drug penetration and bioavailability, thereby providing a method to reduce reported MIC values and improve the potential of THC as an antibacterial agent [20,21]. This experiment revealed that THC exhibited measurable antifungal activity against *Candida albicans* and *Aspergillus niger*; nevertheless, its potency was markedly lower than that of FZ. These findings are preliminary and exploratory, requiring further investigation before considering additional antifungal treatment. The observed dose-dependent inhibition in bacterial and fungal strains may correspond with mechanisms such as membrane disruption or interference with protein synthesis, as reported for other repurposed drugs; however, these remain speculative hypotheses and were not experimentally validated in this study [8,22]. The restricted activity of THC noted in this study suggests that future research could explore its function in combination therapies, where synergistic effects might enhance efficacy or reduce resistance; however, this potential requires validation through synergy studies (e.g., checkerboard or time-kill assays), which were not performed in this investigation. In the contemporary age of AMR, even modestly active molecules might enhance the antibacterial pipeline by serving as adjuncts or scaffolds for chemical improvement [17,23].

Limitations

The in vitro design of this study may not accurately reflect the pharmacokinetics, pharmacodynamics, and host immune interactions that take place in vivo. The antimicrobial testing was limited to a designated panel of bacterial and fungal strains, mainly the American Type Culture Collection (ATCC) reference species, and did not include resistant clinical isolates, which would have enhanced translational relevance. Moreover, the mechanism by which THC exhibits antibacterial properties was not investigated, leading to uncertainty over whether the observed results signify direct microbial suppression or indirect cytotoxicity. This is a substantial limitation, as assessing cytotoxicity is essential before considering THC for antimicrobial repurposing. A further limitation is the lack of post-antibiotic impact studies and time-kill kinetics, which are essential for ascertaining whether THC exhibits bacteriostatic or bactericidal properties. Finally, there was an absence of synergistic tests including regular antibiotics, which could have provided essential insights into the efficacy of combination therapy. Collectively, these limitations emphasize that the current findings should be regarded as exploratory, hypothesis-generating data rather than conclusive evidence of THC's antibacterial potential.

Futuristic approach

Future research must elucidate the molecular mechanisms governing THC's antimicrobial activity, specifically concerning hypotheses such as efflux pump modulation, protein synthesis interference, and microbial membrane disruption; however, these remain conjectural and are not supported by the existing data. Although nanotechnology-based formulations, such as nanogels, nanoparticles, and liposomes, have shown promise in enhancing drug bioavailability and efficacy across different contexts, their application to THC remains speculative, as this study did not yield any formulation or pharmacokinetic data [20,24]. Future research may examine these delivery options to mitigate the limits highlighted below. To ascertain whether THC can augment current treatments and reduce the development of resistance, future research should conduct synergistic trials with standard antibiotics, particularly against multidrug-resistant gram-negative bacteria; however, this potential remains speculative, as synergy testing (e.g., checkerboard or time-kill assays) was not performed in the current study. Verification of its efficacy, tolerability, and pharmacokinetics will need in vivo animal models of infection, thereby connecting in vitro findings to clinical use. The repurposing of THC may aid in the creation of new adjunct therapies to address AMR, hence broadening the treatment pipeline with drugs that have proven safety profiles.

## Conclusions

THC exhibited moderate antimicrobial activity against gram-positive and gram-negative bacteria, as well as fungi; however, its MIC values were consistently higher than those of standard drugs. Despite THC demonstrating lower efficacy than GN and FZ, its activity across various organisms indicates preliminary potential for supplementary antimicrobial strategies; however, this should be approached with caution, as the activity was modest and linked to relatively high MIC values compared to standard agents, rendering its translational significance exploratory at this juncture. Before assessing any therapeutic importance, these findings require validation through mechanistic investigations and synergistic analyses. Nanotechnology-based formulations and in vivo models may provide avenues for future research; nevertheless, they are speculative given this preliminary study which is limited to in vitro testing. Cytotoxicity and pharmacokinetic assessments are essential to determine safety and translational relevance before recommending THC for antimicrobial repurposing. THC may operate as a framework for therapeutic repurposing within the worldwide AMR problem; nonetheless, the current study offers only initial in vitro screening data, lacking mechanistic validation or proof of clinically attainable antimicrobial efficacy. Consequently, its translational potential remains unverified and investigational.
